# An explanation of the limitations relating to ‘An evaluation of the effect of tube potential on clinical image quality using direct digital detectors for pelvis and lumbar spine radiographs’ and the reasoning behind the study – Authors response to letter to the editor

**DOI:** 10.1002/jmrs.425

**Published:** 2020-09-08

**Authors:** Nicole E Peacock, Adam L Steward, Peter J Riley

**Affiliations:** ^1^ Department of Medical Imaging Western Health Footscray Victoria Australia; ^2^ School of Medicine Faculty of Health Deakin University Waurn Ponds Victoria Australia

We wish to thank our colleagues for their interest in our study. Our study is very much set around clinical changes made within our department and as such we fully accept a large number of limitations with it, which we sought to list in the article.^1^ Our study follows a previous basic pilot study that aimed to investigate image acquisition protocols established for film–screen technology as they now relate to digital radiography.^2^ Following this study, our results suggested that there did not appear to exist an overwhelming difference in image contrast between exposures utilising a difference of 15% in kVp. As such, we set out to alter the pre‐set tube potential of a number of projections in our department protocol by increasing these by 15%, expecting that this should reduce the required exposure by around half. Following a trial period of this, we had no adverse feedback from radiologists on the image quality and adopted the practice.

The study data were collected from 3‐ and 6‐month periods, in the middle of which we altered pre‐set tube potentials by 15% and hence we had pelvis images that used 75 kVp and then 85 kVp and lateral lumbar spine images that used 80 and 90 kVp. This study was performed to assess the effect of this clinical change. While for some this is considered an outdated film/screen method of image optimisation, it is still the process most utilised in clinical departments, and as clinical radiographers, the process undertaken at our site and in this study.

Alzyoud *et.al* rightfully question the definition of ‘high tube potential’ and we accept that this term is perhaps misleading, in that our study aimed to differentiate the difference between two tube potentials. We used the term ‘high tube potential’ or ‘high kVp’ as this is how we would tend to clinically refer to the higher of the two assessed tube potentials.

As previously mentioned, the study was a retrospective study conducted following changes made to exposures within our department following a previous pilot study. This provided for us the opportunity to retrospectively assess patients that were imaged immediately prior to, and immediately following, the change of 15% in pre‐set kVp values. This also explains a number of the limitations to our study as we did not directly compare the same patients as the duration of our data collection was over a limited time period. This introduces a number of limitations to our study, which we list in the article. We accept assertions made by Alzyoud and colleagues that using DI introduces a number of ‘frailties’ and we mention this in the article as the DI is usually calculated by either a region of interest or a volume of interest and therefore can vary depending on collimation and patient positioning, among other factors. We have confidence in the consistency of collimation for the pelvis images and routinely, no other protocol variations, such as grid changes, filter use, beam filtration, focal spot size and more are used within our department. We chose to use the DI value as this is the value commonly understood by our colleagues on the clinical floor. Alzyoud et.al also correctly point out the effect of accurate positioning to ensure that the AEC provides correct exposure, citing scoliosis as an example. We did exclude a small number of images that would lead to such aberrations, an example of which Alzyoud *et.al* reference from our article as figure 1B.^1^

Alzyoud *et.al* also raise interest in the variation of the VGA results that exist across the different images in the study and concern at the variation, and however, we are not necessarily surprised by the variation given the varied patient presentations we see within our department, which we have stipulated is a limitation of this retrospective study. This limitation was described in the article, in that we had no control over image acquisition conditions and patient size. We agree that had the study been conducted on an anthropomorphic phantom, that we would expect far greater consistency among the VGA results. As they also suggest, scorer variation merits further evaluation in future studies.

We thank the authors for the opportunity to rescrutinise the data, where it is evident that the average pelvis low kVp DAP is incorrectly reported as 14.06, it should be 11.47 mGy.cm^2^. As requested, the DAP results are here reported as 95% confidence intervals [mean ± 1.96*standard error]:

Pelvis low kVp [11.47 ± 1.77] mGy.cm^2^.

Pelvis high kVp [ 7.47 ± 0.55] mGy.cm^2^.

Lumbar low kVp [15.76 ± 5.25] mGy.cm^2^.

Lumbar high kVp [14.83 ± 3.54] mGy.cm^2^.

Student’s *t*‐tests (two‐tailed, unequal variances) for the Pelvis DAP data show significant difference at p<<0.01, but no significant difference for the Lumbar groups. We did not expect to demonstrate statistically significant differences with such small sample sizes and with such diverse patient presentations, habitus etc. The inclusion of DAP as a simplistic indicator of patient dose was meant to be exploratory with the objective to identify possible trends; the stated differences should be regarded as potential dose savings within the local environment, as previously indicated. Our main statistical concern was to demonstrate that there was no impact on diagnostic outcomes while achieving potential dose reductions at higher kVp.

We also acknowledge the study by Alzyoud *et.al* that concludes that optimal visual grading for pelvis images occurs at 70 and 75 kVp,^3^ and however, the aim of our study was not to identify which of the 2 kVp values assessed were optimal, but rather, to validate that increasing kVp did not significantly degrade the image quality. The study by Alzyoud *et.al* also acknowledge that the higher kVp values provide dose reduction,^3^ which was the impetus for our study. The department protocol at our institution has now permanently changed to the use of the higher kVp values for pelvis and lumbar spine imaging.

We agree with Alzyoud *et.al* that testing a range of patient sizes would be very relevant clinically and a prospective study collecting information on patient weight, BMI and anatomy thickness, as well as investigation into a number of other exposure factors would be very useful. In an ideal prospective study, image acquisition would be standard, on patients able to comply with conventional positioning requirements. We see this study as an assessment of clinical changes made within our department and agree that collaborative multi‐centre studies are the key to building an evidence base for the profession. We see this study as a step in this process; we have found the results of our study to be very exciting as they provide some validation to the clinical outcomes that we have observed locally.

## Conflict of Interest

The authors declare no conflict of interest.
